# Regions of Diversity 8, 9 and 13 contribute to *Streptococcus pneumoniae *virulence

**DOI:** 10.1186/1471-2180-7-80

**Published:** 2007-08-27

**Authors:** Addie Embry, Ernesto Hinojosa, Carlos J Orihuela

**Affiliations:** 1 Department of Microbiology and Immunology, The University of Texas Health Science Center at San Antonio, 7703 Floyd Curl Drive, San Antonio, TX 78229, USA

## Abstract

**Background:**

*Streptococcus pneumoniae *is the leading cause of community-acquired pneumonia. Previously, using comparative genomic analyses, 13 regions of genomic plasticity have been identified in the *S. pneumoniae *genome. These "Regions of Diversity" (RDs) accounted for half the genomic variation observed amongst all pneumococci tested, moreover, were determined to encode a variety of putative virulence factors. To date, genes within 5 RDs have been unequivocally demonstrated to contribute to *S. pneumoniae *virulence. It is unknown if the remaining RDs also contribute to virulence.

**Results:**

Using allelic exchange, we created *S. pneumoniae *mutants that were deficient in RD2, 5, 7, 8, 9, 12 and 13. Mutants deficient in RD8, 9 and 13 were attenuated in a mouse model of disease. RD8 is 40,358 nucleotides in length and encodes 37 genes. Using a panel of isogenic mutants, we determined that RD8b3 is the operon within RD8 that is responsible for virulence. Mice infected with mutants deficient in RD8, RD8b3, RD9 and RD13 had significantly less bacteria in the blood two days after intranasal challenge and improved survival over time versus mice infected with wild type. In all instances mutants colonized the nasopharynx at levels equivalent to wild type.

**Conclusion:**

Genes within RD1, 3, 4, 6, and 10 have previously been shown to contribute to virulence. This study demonstrates that genes within RD8, 9 and 13 also contribute to virulence. The ability of mutants deficient in RD2, 5, 7, 8, 9, 12, and 13 to colonize the nasopharynx indicates that genes within these RDs are not required for asymptomatic carriage. Nonetheless, the observation that mutants deficient in RD8b3, 9 and 13 are attenuated indicates that genes within these loci are necessary for spread of the bacteria beyond the nasopharynx to normally sterile sites.

## Background

*Streptococcus pneumoniae *(the pneumococcus) is a leading cause of community-acquired pneumonia, sepsis, and meningitis. Primarily a commensal, invasive pneumococcal disease (IPD) is characterized by spread of the pneumococcus from the nasopharynx to normally sterile sites such as the lungs, blood, and central nervous system. At risk for IPD are young children, the elderly, and individuals who are immunocompromised or have underlying medical conditions such as sickle cell anemia. Worldwide, it is estimated that *S. pneumoniae *is responsible for 15 cases of IPD per 100,000 persons per year and over a million deaths annually [1,2]. Of note, the preponderance of invasive disease is the result of infection with a relatively few invasive clones [3], a finding that suggests invasive clones carry genes that facilitate disease progression that are absent in non-invasive isolates.

In 2001, the primary nucleotide sequence of three *S. pneumoniae *genomes became publicly available [4-6]. In 2004, comparative genomic hybridization analysis of 10 clinical isolates using microarrays determined that 13 large loci, hence forth termed Regions of Diversity (RD1-13), accounted for half the genomic variation observed among all pneumococci (approximately 10% of the genome of each isolate) [7]. Genomic comparisons, performed by either microarrays or direct sequencing suggested that these 13 RDs are regions of genome plasticity and are unequally distributed among clones and serotype [7,8]. These findings were confirmed by Bruckner et al., who using DNA from 20 *S. pneumoniae *isolates and microarrays, identified 13 clusters of 4 kb and larger that were not shared by a variety of genetically different *S. pneumoniae *strains [9]. Of the 13 RDs, 7 have been associated with atypical GC content and 8 have been determined to be flanked by remnants of mobile genetic elements [7]; findings that indicate these RDs were most likely acquired by horizontal transfer.

To date, 5 RDs have been conclusively demonstrated by deletion mutagenesis to contain genes that contribute to virulence. RD1 encodes ZmpC, a zinc metalloproteinase that plays a role in pathogenicity of the lung [10,11]. RD3 encodes the capsular polysaccharide synthesis operon; capsule is an absolute requirement for virulence [12]. RD4 encodes the pathogenicity islet *rlrA *[13,14]; *rlrA *isresponsible for the formation of pilus-like structures on the surface of the bacteria. Mutants deficient in *rlrA *are attenuated in their ability to adhere to cell lines *in vitro*, colonize the nasopharynx and progress to pneumonia and bacteremia in mice [15]. RD6 encodes Pneumococcal Pathogenicity Island 1 (PPI1) [16]. Virulence genes within PPI1 include, *piaABCD*, an iron acquisition locus, *phgABC*, an operon required for growth in hyperosmotic medium such as blood and serum, and SP1051, a gene with unknown function [16-19]. Finally, RD10 encodes the pathogenicity island *psrP-secY2A2 *[8]. *psrP-secY2A2 *encodes the serine-rich repeat adhesin PsrP. Disruption of *psrP *resulted in a mutant unable to establish lower respiratory tract infection and delayed in its ability to enter the bloodstream. Table 1 describes the 13 RDs identified in *S. pneumoniae*. A list of all the genes within these RDs and their function based on sequence homology has been previously described [8].

**Table 1:** Regions of Diversity in *S. pneumoniae *and their function

Regions of Diversity^†^	% GC content	Genes included in RD: TIGR annotation	Size (Kbp)	Encoded virulence determinants	Ref. cited
*Characterized:*					

RD1	Typical	SP0067-75	10.0	ZmpC; zinc metalloproteinase C	10,11
RD3	Atypical	SP0346-360	16.4	capsular polysacharide synthesis operon	12
RD4	Typical	SP0641-649	18.5	RlrA pathogenicity islet	13–15
RD6	Highly Atypical	SP1028-1065	30.3	PPI1, pneumococcal pathogenicity island I	16–19
RD10	Highly Atypical	SP1755-1772	36.2	PsrP-secY2A2 pathogenicity island	8

*Uncharacterized:*					

RD2	Typical	SP0163-171	6.3	n/a	
RD5	Typical	SP0691-700	4.9	n/a	
RD7	Atypical	SP1129-1147	10.6	n/a	
RD8	Atypical	SP1315-1351	40.3	n/a	
RD9	Typical	SP1612-1622	9.8	n/a	
RD11	Atypical	SP1828-1831	3.7	n/a	
RD12	Highly Atypical	SP1947-1957	11.1	n/a	
RD13	Typical	SP2158-2166	10.9	n/a	

^†^As described by Tettelin et al. [4, 7].

**Figure 1:**
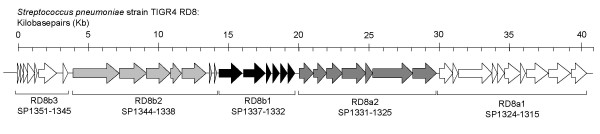
**Schematic representation of RD8**. RD8 is composed of two adjacent pathogenicity islands RD8a and RD8b [8]. Operons within RD8a and RD8b are shown.

In this investigation we examined the remaining uncharacterized RDs (RD2, 5, 7, 8, 9, 11, 12, and 13) to determine if they contribute to virulence. Using allelic exchange we created isogenic deletion mutants deficient in these RDs (with exception to RD11) and determined their ability to colonize the nasopharynx and cause invasive disease in mice. We concluded that genes within RD8, 9 and 13 contributed to *S. pneumoniae *virulence and that none of the RDs examined were required for nasopharyngeal colonization. Finally, RD8 is composed of two pathogenicity islands. These have previously been designated RD8a and RD8b (Figure 1) [8]. We show that genes within the third operon of RD8b, RD8b3, encoded the genes responsible for RD8 virulence.

## Results

### Challenge of mice with *S. pneumoniae *mutants deficient in RD

**Figure 2:**
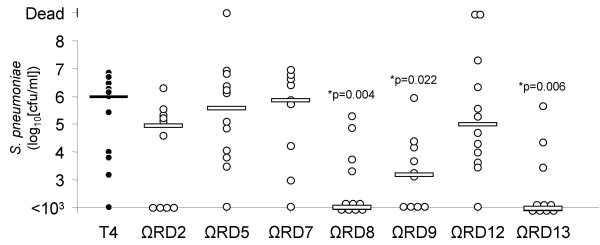
**Bacteria titers in the blood of mice challenged with RD deficient *S. pneumoniae***. Mice were challenged with 10^7 ^cfu of TIGR4 and its isogenic RD mutants. Two days post-challenge, blood was collected from the tail vein of each mouse and the number of bacteria in the blood determined. Each circle represents the bacterial titer of an individual mouse. Bars indicate the median bacterial titer. Statistical analysis was performed using a Mann Whitney Rank Sum Test

**Figure 3:**
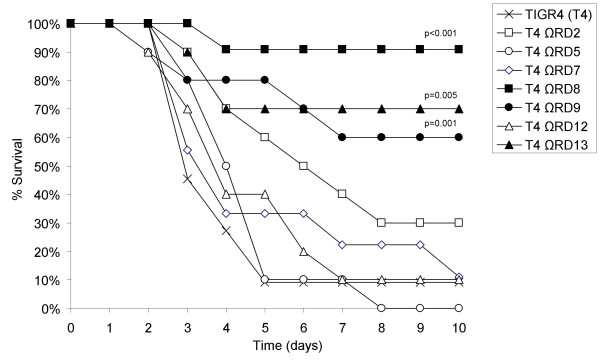
**Percent survival of mice following challenge with RD deficient *S. pneumoniae***. Kaplan-Meier plot illustrating survival of mice infected with wild type and RD deficient mutants. Cohorts of 10–11 mice were infected with wild type and the RD mutants. Survival was recorded over a 10 day period. Statistical analysis was performed using a Gehan-Breslow statistic analysis for survival.

To determine if genes within RD2, 5, 7, 8, 9, 12, and 13 were required for virulence, cohorts of at least 10 BALB/cJ mice were infected intranasally with 10^7 ^cfu of TIGR4 and TIGR4 RD deficient mutants (T4 ΩRD). Mice were monitored for 10 days after challenge and bacterial titers in the blood determined on days 2, 4, 7, and 10. Two days post-challenge bacterial titers in the blood were significantly reduced in mice challenged with T4 ΩRD8, T4 ΩRD9 and T4 ΩRD13 (Figure 2). In contrast, mice challenged with T4 ΩRD2, T4 ΩRD5, T4 ΩRD7, and T4 ΩRD12 had bacterial titers in the blood equivalent to wild type. Mice infected with T4 ΩRD8 and T4 ΩRD9 also had significantly lower bacterial titers on day 4 (T4 ΩRD8: p = 0.008; T4 ΩRD9: p = 0.021), after which an insufficient number of wild type infected mice remained alive for comparison (Figure 3). Of note, significantly lower bacterial titers in the blood at day 2 corresponded with improved survival over time versus infected controls; a finding that indicates genes within RD8, RD9 and RD13 contributed towards virulence.

### Nasopharyngeal colonization

The attenuated phenotype observed with T4 ΩRD8, T4 ΩRD9 and T4 ΩRD13 may be the result of an inability of the mutants to colonize the nasopharynx. To test the contribution of all the RDs to nasopharyngeal colonization, we infected mice intranasally with a sublethal dose of *S. pneumoniae *(10^4 ^cfu) and examined the ability of the mutants to persist in the nasopharynx for up to 2 weeks. No differences were observed in the number of bacteria present in nasopharyngeal lavage elute obtained from mice challenged with the RD mutants and wild type 2, 7, and 14 days after challenge (Table 2). Thus genes within RD2, 5, 7, 8, 9, 12, and 13 did not contribute to nasopharyngeal colonization.

**Table 2:** *S. pneumoniae *titers in nasopharyngeal lavage elute collected from mice

	Day 2			Day 7			Day 14		
Strain	Median (log_10_)	n	p value	Median (log_10_)	n	p value	Median (log_10_)	n	p value

T4	6.41	11		6.30	9		5.25	6	
RD ΩRD2	6.26	9	0.704	5.47	8	0.175	5.39	8	0.662
RD ΩRD5	6.34	10	0.360	6.11	7	0.751	5.46	6	0.589
RD ΩRD7	6.73	9	0.254	5.48	5	0.149	5.11	5	0.537
RD ΩRD8	6.08	8	0.457	6.11	7	0.125	4.87	7	0.295
RD ΩRD9	6.32	8	0.901	5.95	7	0.368	5.30	7	0.628
RD ΩRD12	6.46	10	0.916	6.20	6	0.095	5.68	6	0.240
RD ΩRD13	6.38	7	0.556	6.30	7	0.397	4.96	7	0.945

### Challenge of mice with *S. pneumoniae *mutants deficient in RD8 operons

**Figure 4:**
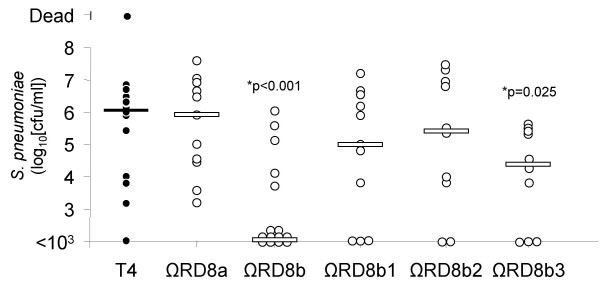
**Bacteria titers in the blood of mice challenged with isogenic mutants of RD8**. Mice were challenged with 10^7 ^cfu of TIGR4 and isogenic mutants deficient in operons within RD8. Two days post-challenge, blood was collected from the tail vein and the number of bacteria in the blood determined. Each circle represents the bacterial titer of an individual mouse. Bars indicate the median bacterial titer. Statistical analysis was performed using a Mann Whitney Rank Sum Test

**Figure 5:**
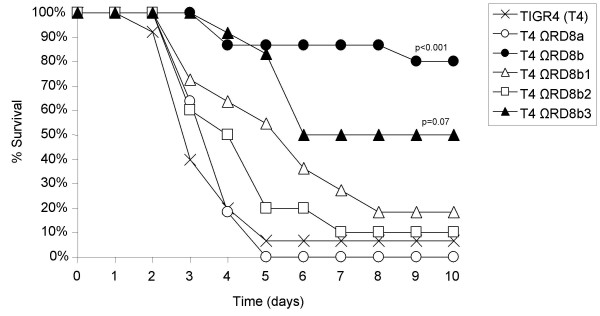
**Percent survival of mice following challenge with RD8 mutants**. Kaplan-Meier plot illustrating survival of mice infected with wild type and RD8 operon deficient mutants. Cohorts of 11–12 mice were infected with wild type and the RD8 mutants. Survival was recorded over a 10 day period. Statistical analysis was performed using a Gehan-Breslow statistic analysis for survival.

RD8 is greater than 40,000 nucleotides in length and encodes 37 genes divided into 5 operons (Figure 1). Previously, using comparative genomic analyses, we determined that RD8 is composed of two pathogenicity islands, RD8a and RD8b, that are adjacent to each other on the TIGR4 chromosome [8]. To determine which of these pathogenicity islands were required for virulence we created two mutants, one deficient in RD8a, which lacked the genes SP1315 to SP1331, and one deficient in RD8b which lacked the genes SP1332 to SP1351, and tested the ability of these mutants to cause disease. Two days post-challenge mice infected with T4 ΩRD8b had bacterial titers in the blood significantly lower than mice infected with TIGR4, whereas mice infected with T4 ΩRD8a had bacterial titers in the blood comparable to wild type (Figure 4). As with T4 ΩRD8, lower bacterial titers of T4 ΩRD8b in the blood corresponded with improved survival (Figure 5). All mice infected with RD8a died within 5 days of challenge (n = 11), whereas 91% (n = 11) of those infected with T4 ΩRD8b survived past day 10.

To determine the operon(s) within RD8b that were responsible for virulence, we created isogenic mutants deficient in the operons RD8b1, RD8b2 and RD8b3 and challenged mice with these mutants. Deletion of RD8b1 or RD8b2 failed to have a statistically significant impact on virulence. Mice infected with T4 ΩRD8b1 and T4 ΩRD8b2 had bacterial titers in the blood equivalent to wild type at day 2 and a survival rate comparable to wild type infected mice (Figure 4 and 5). In contrast, deletion of RD8b3 attenuated TIGR4 virulence. Mice infected with T4 ΩRD8b3 had 40-fold less bacteria in the blood than mice infected with TIGR4 (Figure 4), and demonstrated improved survival over time (Figure 5). Thus genes within RD8b3 were in large part responsible for the virulence associated with RD8.

Unlike deletion of RD8b1-3 (T4 ΩRD8b), it is of note that deletion of RD8b3 did not completely attenuate TIGR4 virulence. Moreover, the mutant T4 ΩRD8b1 was not fully virulent. At day 2 mice infected with T4 ΩRD8b1 had a median bacterial titer in the blood 10-fold lower than wild type (p = 0.337), likewise, mice infected with T4 ΩRD8b1 demonstrated a delay in time of death (p = 0.09), albeit these differences were not significant. Thus it seems that genes within RD8b1 might also contribute to virulence, but not in a manner sufficient to demonstrate statistical significance in this challenge model.

### TIGR4 growth in media with ribulose and fucose as the sole carbon source

Based on nucleotide sequence homology, genes within RD9 and RD13 encode proteins that transport ribulose and fucose, respectively (see Discussion). To test this prediction, we determined if deletion of RD9 and RD13 affected the ability of TIGR4 to grow in media containing either ribulose or fucose as the sole carbon source. In all instances TIGR4, T4 ΩRD9 and T4 ΩRD13 grew normally in regular C+Y (0.15% glucose and 0.2% sucrose; data not shown). However, neither TIGR4 nor the mutants were able to grow in media containing ribulose (0.1–0.5%) or fucose (0.1–0.5%) as the sole carbon source (data not shown). Thus ribulose or fucose is not sufficient to support TIGR4 growth despite the presence of RD9 and RD13.

## Discussion

It is well established that the propensity of a *S. pneumoniae *isolate to cause invasive disease is dependent on the interplay between serotype and genotype. Molecular epidemiology has demonstrated that invasive and non-invasive clones exist within invasive serotypes [20]. Kelly et al. have shown that conversion of a virulent serotype 5 isolate to serotype 3 ablated virulence in mice, whereas conversion of a 6B isolate to serotype 3 lowered the LD_50 _by >100-fold [21]. It is also known that the genomic component of virulence (i.e. that excluding genes that encode capsular polysaccharide) is dependent on the presence of horizontally acquired genes that complement the core genome. Previously, we have demonstrated that RDs known to contribute to virulence are unequally distributed among invasive and non-invasive isolates in a serotype-dependent manner [8]. Likewise, the absence of non-core, non-RD associated virulence genes, such as those encoding the adhesin Choline Binding Protein A (CbpA), have been demonstrated to diminish the ability of an isolate to progress from the nasopharynx to the lungs and bloodstream [22].

To date, RD1, 3, 4, 6 and 10 have been conclusively shown to contribute to virulence by deletion and disruption mutagenesis [8,10-16,18,19,23]. The purpose of this study was to determine if the 8 uncharacterized RDs (RD2, 5, 7, 8, 9, 11, 12 and 13) also contribute to virulence. Because all 13 RDs are present in TIGR4 [4], we chose to use it as the parent strain for these analyses.

The fact that we were able to create deletion mutants deficient in RD2, 5, 7, 8, 9, 12 and 13, moreover, that these mutants colonized the nasopharynx of mice normally, indicates that genes within these RDs were not essential *in vitro *nor required *in vivo *by TIGR4 for asymptomatic carriage. Given that the human nasopharynx is the sole ecological niche of *S. pneumoniae*, this finding is consistent with the fact that RDs are not present in all clinical isolates and serve as accessory genes. Notably, we were unable to create a mutant deficient in RD11 despite several attempts. RD11 encodes a protein homologous to the high affinity phosphate transport system (PstSABC) regulatory protein, PhoU. Studies have shown that PstSABC is expressed in response to low levels of phosphate and that deletion of PhoU in strain R6X has pleiotropic effects including delayed growth and an inability to undergo autolysis [24,25]. Thus, one possible explanation for our inability to create an RD11 mutant is that its deletion negatively impacts the ability of TIGR4 to acquire phosphate.

Challenge of mice with the panel of RD mutants demonstrated that genes within RD8b3, 9 and 13 were required by TIGR4 to cause invasive disease. Given that mutants deficient in these RD colonized the nasopharynx normally, it can be inferred that these loci encode virulence determinants that are not required for colonization but instead facilitate spread of the bacteria to normally sterile sites. How these loci contribute to virulence remains to be determined.

RD8 is composed of two pathogenicity islands located adjacent to each other on the TIGR4 chromosome. Previously, using comparative genomic analysis, we determined that the presence of RD8a was positively correlated with the ability of serotype 6A and 6B clinical isolates to cause invasive human disease. Likewise, we determined that genes within RD8b were correlated with the non-invasive phenotype [8]. In this manuscript we demonstrate that deletion of RD8a has no impact on TIGR4 virulence, whereas deletion of RD8b ablates TIGR4 virulence. Thus a discrepancy exists between the findings of this study and those from our previous comparative genomic analysis. One explanation for this discrepancy may be the differences in strains. Comparative genomic analyses was performed using 72 clinical isolates of serotype 6A, 6B and 14, whereas this study was limited to a single serotype 4 isolate. Thus it is possible that genotype (i.e. gene redundancy, transcriptional regulation, etc...) and serotype dependent factors (i.e. resistance to opsonophagocytosis, ability to adhere to cells) have a large impact on the requirement of RDs for virulence. This would be in accordance with the findings reported by Kelly et al. that are described above, moreover, explain why RDs known to be required for virulence in one strain are not found in others. For example zmpC, a zinc metalloproteinase encoded within RD1, is present in only 25% of clinical isolates [26]. This finding also suggests that it is not possible to evaluate the role of any single gene or operon in any single strain or serotype.

### Possible roles of RD8b3, RD9 and RD13

The operon RD8b3 is composed of 7 genes: 6 small hypothetical genes (SP1345, SP1347-49, SP1351), a conserved domain protein (SP1350), and a putative membrane protein (SP1346). BLASTP analysis of the predicted amino acid sequence of these genes finds homologues in other streptococci and gram-positive bacteria, but no indication of function with exception to SP1346. SP1346 is identified as a member of the CAAX amino terminal protease family. Members of this family include the bacteriocin-like immunity protein PlnP from *Lactobacillus plantarum *[27]. PlnP mediates protection against highly charged cationic proteins that are expressed from the same operon. Interestingly, SP1345, SP1347, SP1350 are less than 150 amino acids in length and have a pI greater than 9. SP1349 and SP1351 are less than 100 amino acids in length have a pI less than 4. Thus, SP1346 may be an immunity-like protein that protects the bacteria from cationic and anionic peptides (presumably the hypothetical proteins), that may serve to impair the host defence. Future studies are warranted to determine if this is the case.

RD9 encodes a single operon composed of 11 genes. It encodes a transketolase, a ribulose phosphate 3-epimerase, a sugar phosphotransferase (PTS) system, and a transcriptional terminator. RD13 is also a single operon composed of 9 genes. It encodes an L-fucose isomerase, a fucolin-related protein, an L-fucose aldolase, a PTS transport system, and a conserved hypothetical protein. Thus RD9 and RD13 encode genes that may contribute to ribulose and fucose uptake.

In context with what is known in regards to RD6 (iron uptake) [18,19,23] and RD11 (phosphate uptake) [24], the finding that mutants deficient in RD9 and RD13 are attenuated suggests that acquisition of ribulose and fucose are essential for TIGR4 during the disease process. Unfortunately, TIGR4 failed to grow in media that contained ribulose or fucose as the sole carbon source. This finding brings up the possibility that: 1) ribulose and fucose play a role in secondary metabolism, which indirectly contributes to virulence, but alone are not sufficient to support bacterial growth. 2) That these RDs do not transport sugars and contribute to virulence in an undefined manner. As with RD8b3, future experiments are warranted to determine the mechanism by which these loci contribute to virulence.

## Conclusion

The goal of this study was to determine the virulence contribution of the 8 uncharacterized *S. pneumoniae *RDs. Having done so, we can now focus future efforts on characterization of genes within RD8b3, RD9 and RD13. Future studies will focus on the role RD8b3 as a potential bacteriocin-like system that mediates virulence, and the requirement for fucose and ribulose *in vivo*. Of the 13 *S. pneumoniae *RDs identified by Tettelin et al., and Bruckner, seven have now been conclusively demonstrated to play a role in virulence. This strongly suggests that the propensity of an isolate to cause invasive disease is dependent on the presence of horizontally acquired genes that complement the core virulence genes present.

## Methods

### Bacterial strains and components

*S. pneumoniae *serotype 4, strain TIGR4 [4] and its isogenic mutants were grown in defined semi-synthetic casein liquid media supplemented with 0.5% yeast extract (C+Y) [28] and tryptic soy blood agar plates (Remel, Lenexa, KS) at 37°C in 5% CO_2_. Mutants with deleted RDs were grown with erythromycin at 1 μg/ml (Sigma-Aldrich, St. Louis, MO). *Escherichia coli *TOP10 cells (Invitrogen, Carlsbad, CA) containing the vector pCR2.1 (Invitrogen) and subsequent recombinant plasmids were selected on Luria Bertani agar plates containing 1 mg/ml erythromycin. Standard molecular techniques were used for plasmid DNA manipulation and transformation of *E. coli *[29].

### Creation of Region of Diversity (ΩRD) mutants

Mutants were created using a modified version of the SOEing technique [30]. The erythromycin resistance cassette *ermB *was PCR amplified from plasmid pTCV-lac [31] and cloned into the TA cloning vector pCR2.1. DNA fragments representing sequences immediately 5' and 3' of the RDs targeted for deletion were amplified from TIGR4 genomic DNA and cloned upstream and downstream of the *ermB *cassette in pCR2.1. Primers used to amplify the upstream and downstream fragments were designed to include restriction sites that allowed direction cloning of the fragments. Fragments were cloned such that after transformation, transcription of *ermB *was antisense to that of surrounding genes. Once cloned in *Escherichia coli*, mutagenic PCR products (~3 kb) containing the *ermB *cassette and the two flanking fragments were amplified from purified plasmid and the DNA construct used to transform TIGR4. Transformation was performed using CSP-2 following standard methods [32]. Erythromycin-resistant clones were selected on tryptic soy blood agar plates containing 0.5 μg/ml of erythromycin. Deletions were confirmed by 1) successful amplification of a PCR product using primers that flanked the deleted loci, 2) sequencing of these PCR products, and 3) a failure to amplify genes located within the deleted RD from chromosomal DNA isolated from the mutants. Table 3 lists the primers used to create the mutagenic and the genes deleted as the result of allelic exchange.

**Table 3:** PCR primers used to create T4ΩRD mutants and the corresponding genes deleted

	Genes deleted: TIGR annotation	Primers used to amplify flanking DNA fragments for PCR construct deletion mutagenesis†	
Strain		Upstream fragment	Downstream fragment

T4 ΩRD2	SP0163-0168	SP0162 F: NNNNNaagcttGGGATTGGGCTCCTATG	SP0168 F: NNNNNgcggccgcGTACCAACAAATACCTC
		SP0163 R: NNNNNactagtCTGCTAAAGATAGG	SP0170 R: NNNNNtctagaTCTAACTCTTCATAG
T4 ΩRD5	SP0694-0700	SP0694 F: NNNNNaagcttCCTATCTTGTTGTCATTA	SP0698 F: NNNNNgcggccgcATGGGAACTACTCC
		SP0694 R: NNNNNactagtCGTAGAACATATTTCGTCC	SP0800 R:NNNNNtctagaACCCAAAACTTCTGC
T4 ΩRD7	SP1130-1145	SP1130 F: NNNNNaagcttCAATTTCTGAAGGTCCG	SP1145 F: NNNNNgcggccgcCATACTGATGATGAGG
		SP1130 R: NNNNNactagtGTTTAAGCCATGGCG	SP1146 R: NNNNNtctagaCCATAACTAACTCTCC
T4 ΩRD8	SP1315-1352	SP1315 F: NNNNNgcggccgcACTTCGGAAAGAAGTGG	SP1352 F: NNNNNaagcttGTTTCAATGCCCAGCTTCGTCC
		SP1315 R: NNNNNtctagaCTTCACTTTCATAATACG	SP1352 R: NNNNNactagtCTTTAATGCATCATTAACGACGC
T4 ΩRD8a	SP1315-1331	SP1315 F	SP1331 F: NNNNNaagcttTGTTACTGCAAAAAGAAC
		SP1315 R	SP1331 R: NNNNNactagtGCGCCTTTATCTGCAGC
T4 ΩRD8b	SP1332-1352	SP1332 F: NNNNNgcggccgcGAATTTCTTTGTATCGG	SP1352 F
		SP1332 R: NNNNNtctagaGCAGCAAACTATAAAC	SP1352 R
T4 ΩRD8b1	SP1332-1337	SP1332 F	SP1337 F: NNNNNaagcttCAGTTTACCAAATCATC
		SP1332 R	SP1337 R: NNNNNactagtCTGCCTCTTCAGAACAATAACG
T4 ΩRD8b2	SP1338-1344	SP1339 F: NNNNNgcggccgcTGCGGTAACTGGAGTG	SP1344 F: NNNNNaagcttTAAAATATACTGGCACGG
		SP1338 R: NNNNNtctagaTTCTTCTCTACAAGCTC	SP1344 R: CGATGTAGTTactagtGCC
T4 ΩRD8b3	SP146-1352	SP1346 F: NNNNNgcggccgcCCAACAAGTAAATGTCC	SP1352 F
		SP1346 R:NNNNNtctagaTATAGCGCACCATACC	SP1352 R
T4 ΩRD9	SP1615-1621	SP1615 F: NNNNNaagcttGGGACTTAACTTGAGC	SP1621 F: NNNNNgcggccgcATAGGGAATGTTTACCC
		SP1615 R: NNNNNactagtCCATTCAGGAGTTGCC	SP1621 R: NNNNNtctagaAAACAAGAAC
T4 ΩRD12	SP1950-1957	SP1950 F: NNNNNaagcttACGAGTGGATTGAC	SP1957 F: NNNNNgcggccgcGATTGAACTTAAACAGG
		SP1950 R: NNNNNactagtCTTCATAGCTGTGATCCG	SP1957 R: NNNNNtctagaGCCATCTCCCAAATTGCC
T4 ΩRD13	SP2158-2166	SP2158 F: NNNNNaagcttGGAATTCTCAACATAG	SP2166 F: NNNNNgcggccgcCATCTCCTCATCAGG
		SP2158 R: NNNNNactagtCCACAAATCTTTGC	SP2166 R: NNNNNtctagaCAGATTTGACGAAAGG

### Intranasal challenge model

Five week old female BALB/cJ mice (Jackson Laboratory, Bar Harbor, ME) were maintained in biosafety level 2 facilities at The University of Texas Health Science Center in San Antonio. All experiments were performed with mice under general anaesthesia with inhaled isoflurane (2.5%; Baxter Healthcare Corp., Deerfield, IL). *S. pneumonia*, 10^4 ^cfu (low dose colonization model) or 10^7 ^cfu (invasive disease model) in 20 μl phosphate buffered saline (PBS) was administered drop-wise to the external nares of mice. On day 2, 4, 7 and 10 bacterial titers in the blood were determined by collection of blood from the tail vein, serial dilution and plating. Bacterial titers in the nasopharynx were determined on days 2, 7, and 14 by serial dilution and plating of nasopharyngeal lavage obtained by instillation of 20 μl PBS into the right nostril and collection of elute from the left. Statistical analysis of bacterial titers was performed using a nonparametric independent group analysis (Mann-Whitney rank sum).

### Ribulose and fucose growth curves

Exponential phase cultures (OD_620 _= 0.5) of TIGR4, T4 ΩRD9 and T4 ΩRD13 were grown in C+Y, centrifuged, washed, and suspended in PBS. Borosilicate glass tubes (15 × 100 mm) containing 5 ml of normal C+Y (0.15% glucose, 0.2% sucrose), C+Y with ribulose as the sole carbon source (0.1–0.5% fucose), and C+Y with fucose as the sole carbon source (0.1–0.5% fucose) were inoculated with 100 μl of bacterial suspensions (final concentration: 10^5 ^cfu/ml) and grown at 37°C in 5% CO_2_. Microbial growth was measured by determining the optical density of the culture at OD_620 _on an hourly basis for 6 hours. Three cultures were measured at each time point for each of the bacteria tested.

### Data analysis

Statistical analysis of mouse survival over time was performed using a Gehan-Breslow statistic analysis for survival. Statistical analysis of bacterial titers in the blood and nasal lavage was performed using a Mann-Whitney Rank Sum Test.

## Authors' contributions

AE created the RD mutants used in these studies and contributed to the writing of the manuscript. EH was responsible for the experiments in mice. CJO conceived the study, oversaw all aspects of the investigation, performed the statistical analysis, and drafted the manuscript. All authors read and approved the final manuscript.
